# Abnormal percent amplitude of fluctuation changes in patients with monocular blindness: A resting-state functional magnetic resonance imaging study

**DOI:** 10.3389/fpsyt.2022.942905

**Published:** 2022-10-24

**Authors:** Qiaohao Hu, Jun Chen, Min Kang, Ping Ying, Xulin Liao, Jie Zou, Ting Su, Yixin Wang, Hong Wei, Yi Shao

**Affiliations:** ^1^Department of Ophthalmology, The First Affiliated Hospital of Nanchang University, Jiangxi Branch of National Clinical Research Center for Ocular Disease, Nanchang, China; ^2^Department of Ophthalmology and Visual Sciences, The Chinese University of Hong Kong, Shatin, Hong Kong SAR, China; ^3^Department of Ophthalmology, Massachusetts Eye and Ear, Harvard Medical School, Boston, MA, United States; ^4^School of Optometry and Vision Sciences, College of Biomedical and Life Sciences, Cardiff University, Cardiff, United Kingdom

**Keywords:** MB, PerAF, resting state, fluctuation changes, functional magnetic resonance imaging

## Abstract

**Purpose:**

Previous studies on monocular blindness (MB) have mainly focused on concept and impact. The present study measured spontaneous brain activity in MB patients using the percentage of amplitude fluctuation (PerAF) method.

**Methods:**

Twenty-nine patients with MB (21 male and 8 female) and 29 age-, gender-, and weight-matched healthy controls (HCs) were recruited. All participants underwent resting state functional magnetic resonance imaging (rs-fMRI). The PerAF method was used to analyze the data and evaluate the spontaneous regional brain activity. The ability of PerAF values to distinguish patients with MB from HCs was analyzed using receiver operating characteristic (ROC) curves, and correlation analysis was used to assess the relationship between PerAF values of brain regions and the Hospital Anxiety and Depression Scale (HADS) scores.

**Results:**

PerAF values in Occipital_Mid_L/Occipital_Mid_R/Cingulum_ Mid_L were significantly lower in patients with MB than in controls. Conversely, values in the Frontal_Sup_Orb_L/Frontal_Inf_Orb_L/Temporal _Inf_L/Frontal_Inf_Oper_L were significantly higher in MB patients than in HCs. And the AUC of ROC curves were follows: 0.904, (*p* < 0.0001; 95%CI: 0.830–0.978) for Frontal_Sup_Orb_L/Frontal_Inf_Orb_L; Temporal_Inf_L 0.883, (*p* < 0.0001; 95% CI: 0.794–0.972); Frontal_Inf_Oper_L 0.964, (*p* < 0.0001; 95% CI: 0.924–1.000), and 0.893 (*p* < 0.0001; 95% CI: 0.812–0.973) for Occipital_Mid_L; Occipital_Mid_R 0.887, (*p* < 0.0001; 95% CI: 0.802–0.971); Cingulum_Mid_L 0.855, (*p* < 0.0001; 95% CI: 0.750–0.960).

**Conclusion:**

The results of our study show abnormal activity in some brain regions in patients with MB, indicating that these patients may be at risk of disorder related to these brain regions. These results may reflect the neuropathological mechanisms of MB and facilitate early MB diagnoses.

## Introduction

Vision loss, including blindness, is a major public health problem, affecting individual lives, society, and the economy. Visual impairment has negative impacts on standard of living and self-care, is a heavy social burden, and may cause significant economic damage. Traditionally, the definition of blindness has been based on functional disability and quantized visual acuity (VA) value. However, the diagnostic criteria for blindness vary between countries (1). Many factors can cause blindness, including diseases, such as age-related macular degeneration (AMD) (2), cataract (3), trachoma (4), and glaucoma (5). Among them, AMD is the most important cause of blindness (2). Although there are treatments for these diseases, if they are not addressed promptly or effectively, they can cause irreversible damage to visual function and can eventually lead to blindness. The World Health Organization (WHO) estimated that about 1.3 billion people may suffer from visual impairment (VI) globally, with a range of causes (6). According to one study, it is estimated that worldwide in 2015 about 36 million people were blind, 216.6 million people suffered from moderate to severe VI, while 185.5 million people had mild VI (7). These data show that VI affects many people and this should arouse public attention. Blindness may be unilateral (monocular) or bilateral, and its incidence is related to factors such as heredity and environment. This paper focuses on monocular blindness (MB).

Monocular blindness is defined as the reduction or loss of visual input caused by the damage or destruction of retina or optic nerve. The naked visual acuity of blind eye is <0.05, and the naked visual acuity of contralateral eye is ≥0.05. Studies have shown that MB may occur at any age and affect either gender (8), and includes the loss of stereo vision, perception of shape and color and other visual functions (9). It should be noted that progression may continue beyond monocular blindness, and that if the other eye is not treated properly both eyes may become affected (10). Therefore, once MB is diagnosed the cause should be treated without delay to prevent the adverse consequences of binocular blindness.

The etiology of MB is varied, eye trauma being a major cause in children (11). In developing countries, adult cataracts and glaucoma are important causes (12–14), while in developed countries AMD and diabetic retinopathy are major causes (15).

As a non-invasive method, functional magnetic resonance imaging (fMRI) can evaluate brain structure and function, and researchers have found associations between fMRI measurement and clinical manifestations of diseases (16). Compared with other neuroscience technologies, the advantage of fMRI lies in its flexibility (17). Multi-level brain imaging analysis is an effective means to find brain structural and functional changes in early postoperative chronic disease. Yang used fMRI technology to explore the immediate brain effect of Moxibustion in patients with primary dysmenorrhea and found that positive activation was the main manifestation of local consistency and coordination in the brain area of patients, and the prefrontal lobe was likely to play an analgesic and sedative role. The change of prefrontal lobe-default network (DMN) functional connection may be an important central mechanism of analgesia (18).

Resting state functional magnetic resonance imaging (rs-fMRI) depends on the spontaneous low-frequency fluctuations in the blood oxygen level-dependent (BOLD) signal with non-invasive, zero radiation and high spatial resolution has become an effective new means of contemporary acupuncture research (19), which can objectively and visually observe the central functional regulation of the brain. Regional Homo geneity(Re HO),functional connection(FC), and fractional amplitude of low frequency fluctuation(fALFF) are the two most commonly used data analysis methods in rs-fMRI (20). The former represents the consistency of local functional activities in brain regions and reflects the synchronization of time series in local brain regions (21). The latter represents the strength of functional connection between brain regions (22), and can reflect the temporal correlation between region of interest (ROI) and whole brain networked function, fALFF has high sensitivity and specificity, and can accurately reflect the spontaneous neuronal activity in low-frequency local brain areas (23).

The peraf value is less affected by the error of signal intensity, and can be used for group level statistical analysis (24). It is not affected by the mixing of voxel specific fluctuation amplitude in the amplitude of low-frequency fluctuation method. It can measure the brain activity change of voxel level more accurately and efficiently (25). It is more accurate than other MRI analysis methods such as low-frequency fluctuation amplitude (26), regional homogeneity and degree centrality (27). It is of great significance for the diagnosis and treatment of monocular blindness (28).

After monocular blindness, one side of the visual function is normal, and the other side is damaged. At this time, the brain’s visual reflex mechanism may increase in the normal side, and thus peraf changes. Another is the disorder of visual transmission mechanism in patients with monocular blindness. At this time, the brain may not be stimulated by visual activity, and the function of the corresponding parts will be reduced, resulting in changes in peraf (29). The peraf value is less affected by the error of signal intensity, and can be used for group level statistical analysis. It is not affected by the mixing of voxel specific fluctuation amplitude in the amplitude of low-frequency fluctuation method. It can measure the brain activity change of voxel level more accurately and efficiently. It is more accurate than other MRI analysis methods such as low-frequency fluctuation amplitude, regional homogeneity and degree centrality. It is of great significance for the diagnosis and treatment of monocular blindness (30).

The present study will apply percentage of amplitude fluctuation (PerAF) technology to study the spontaneous brain regional activity and clinical manifestations of MB patients, and to investigate whether this method can be used for early diagnosis of MB.

## Materials and methods

### Patients

Twenty-nine patients with MB (21 male and 8 female) were recruited at the Ophthalmology Department of the First Affiliated Hospital of Nanchang University. These subjects satisfied the following criteria: (1) blind in one eye; (2) contralateral eye is normal without cataract, optic neuritis, or other eye diseases; (3) exclude strabismus.

In addition, 29 healthy controls (21 male and 8 female) were recruited and the two groups were similar in gender balance (*p* > 0.99), age (*p* = 0.792), and weight (*p* = 0.881). Control subjects were included if they satisfied the following criteria: (1) normal naked eye or normal corrected vision; (2) no neurological diseases; (3) no mental disorder; (4) able to have an MRI scan (for example, they did not have pacemaker or implanted metal device) (Table 1).

**TABLE 1:** Characteristics of participants included in the study.

Condition	MB	HCs	t	*P*-value*
Male/female	21/8	21/8	N/A	>0.99
Age (years)	47.76 ± 6.76	46.23 ± 6.61	0.168	0.792
Weight (kg)	71.87 ± 5.98	72.71 ± 6.87	0.286	0.881
Handedness	29R	29R	N/A	>0.99
Duration of MB (h)	58.54 ± 25.54	N/A	N/A	N/A
Best-corrected VA-left eye	0.25 ± 0.10	1.05 ± 0.15	−5.965	0.002
Best-corrected VA-right eye	0.20 ± 0.05	1.15 ± 015	−5.653	0.003

*p* < 0.05 independent *t*-tests comparing two groups, data shown as mean ± standard deviation. MB, monocular blindness; HCs, healthy controls; VA, visual acuity; N/A, not applicable.

The research was authorized by the Human Research Ethics Committee of the First Affiliated Hospital of Nanchang University. Each participant understood the aim, methods and possible risks of the research, and signed a declaration of informed consent.

### Magnetic resonance imaging data collection

The Trio 3-Tesla MR scanner (Siemens, Munich, Germany) was used. Before scanning, each participant was asked to relax, close their eyes, and minimize movement (29). To obtain functional data, a 3D metamorphic gradient echo pulse sequence was used. The following parameters were used for a 176-image scan: acquisition matrix 256 × 256; field of view 250 mm × 250 mm; echo time 2.26 ms; repetition time 1,900 ms; thickness 1.0 mm; gap 0.5 mm; flip angle 9°. For a 240-image scan, parameters were as follows: acquisition matrix 64 × 64; field of view 220 mm × 220 mm; thickness 4.0 mm; gap 1.2 mm; repetition time 2,000 ms; echo time 30 ms; flip angle. 90°, 29 axial.

### Functional magnetic resonance imaging processing

MRIcro software (Nottingham University, Nottingham, UK) was used to sort the data, and to identify and exclude incomplete or flawed data. Remaining data were processed, including space standardization, head movement correction, slice time, and digital image format conversion using DPARSFA.^1^ Linear regression was used to eliminate the influence of factors such as signals originating from white matter.

Because excessive head movement may have a significant impact on the fMRI sequence, participants with head movements >3 mm and the data were excluded. Due to inter-individual variations in brain size and structure, each brain image was standardized (30). We used regions of interests (ROI) of the central white matter region to deal with irrelevant variables (31).

Functional magnetic resonance imagingdata were processed using the PerAF method, a relatively reliable and direct measurement of brain activity. First, the average BOLD signal value was calculated, then the signal strength at a range of time points was normalized to this value. This process resulted in an amplitude at each time point as a percentage of the average across the time series, and a signal change percentage similarity index, referred to as PerAF. The formula used to calculate the PerAF value of a single voxel is as follows:


(1) PerAF= 1⁢n⁢Σ⁢i= 1⁢n⁢|Xi-μ⁢μ|× 100%

(2) μ= 1⁢n⁢Σ⁢i= 1⁢nXi

Where Xi represents the signal strength, n is the total number of time points, and μ is the mean value of the time series (21).

### Correlation analysis

We obtained the anxiety scores (AS) and depression scores (DS) of MB patients by doing the Hospital Anxiety and Depression Score (HADS). We looked for correlations between each score and the PerAF values of the following brain regions: Frontal_Sup_Orb_L/Frontal_Inf_Orb_L, and Frontal_Inf_Oper_L using Pearson’s correlation analysis (*p* < 0.05 was considered significant). GraphPad Prism 8.0 software was used to plot linear correlations.

### Statistical analysis

For between-group comparisons, SPSS software, version 20.0 (IBM Corp., Armonk, NY, USA) was used to conduct independent sample t tests, and *p* < 0.05 was considered significant. The REST software was used to conduct independent sample t tests comparing PerAF values between the two groups. Gaussian random field theory was used for multiple comparison correction, and the voxel level threshold was *p* < 0.001. AlphaSim, part of the REST toolbox, was used for correction, the cluster size was set at >49 voxels, and the level was *p* < 0.05. Receiver operating characteristic (ROC) curves were used to compare the average PerAF values of the relevant brain areas between MB and HC groups and to obtain estimates of diagnostic accuracy based on the area under the curve (AUC). As explained above, Pearson’s correlation was used to evaluate the relationship between PerAF and anxiety/depression scores. All averaged data are presented in the form of mean ± standard deviation. The regions were defined using automatic anatomic labeling based on the Montreal Neurological Institute data set.

## Results

### Sample statistic and visual data

Gender (*p* > 0.99), age (*p* = 0.792), and weight (*p* = 0.881) were all similar in the two groups. However, significant differences were found between groups in monocular best-corrected visual acuity (VA) (left *P* = 0.002; right *p* = 0.003). The duration since MB diagnosis was 58.54 ± 25.54 h.

### Percentage of amplitude fluctuation differences

**FIGURE 1:**
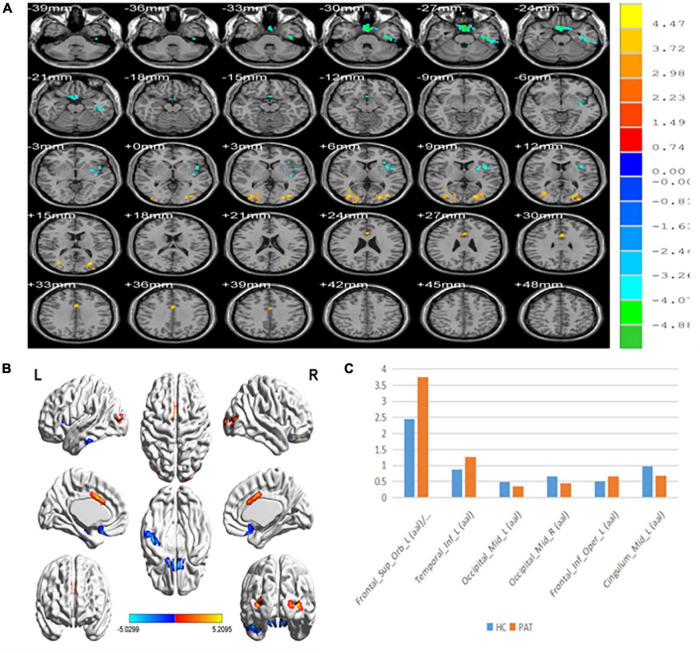
**(A,B)** Spontaneous brain activity in the MB patients and the HC group. **(C)** The mean PerAF signal value between the MB and HC groups. Warmer shades (yellow and red) represent moderate and high signal strength, respectively and blue represents lower signal strength. The signal values of Frontal_Sup_Orb_L/Frontal_Inf_Orb_L/Temporal_inf_L/Frontal_Inf_Oper_L regions in MB patients are higher than in controls, and on the contrary, the signal valuse of Occipital_Mid_L/Occipital_Mid_R/Cingulum_Mid_L are lower than controls. PerAF, percent amplitude of fluctuation; MB, monocular blindness; HCs, healthy controls.

Compared with HCs, PerAF values were significantly reduced in MB patients at the Occipital_Mid_L/Occipital _Mid_R/Cingulum_Mid_L. Conversely, values were significantly higher in MB than HC at the Frontal_ Sup_Orb_L/Frontal_Inf_Orb_L/Temporal_Inf_L/Frontal_Inf_ Oper_L. (Table 2 and Figure 1).

**TABLE 2:** Brain areas with significantly different PerAF values between MB and HCs.

Brain areas	MNI coordinates			Number of voxels	T value	ROI
	X	Y	Z			
**HCs < MB**						
Frontal_Sup_Orb_L/Frontal_Inf_Orb_L	0	18	−27	169	−5.0299	1
Temporal_Inf_L	−42	−18	−27	98	−4.917	2
Frontal_Inf_Oper_L)	−39	15	9	111	−4.4132	5
**HCs > MB**						
Occipital_Mid_L	−36	−81	3	112	5.2095	3
Occipital_Mid_R	36	−81	6	112	4.5945	4
Cingulum_Mid_L	−3	9	33	50	4.5309	6

The statistical threshold was set at the voxel level with *p* < 0.001 for multiple comparisons using Gaussian random field theory (*p* < 0.01, cluster >49 voxels, AlphaSim corrected). PerAF, percent amplitude of fluctuation; ROI, regions of interest; HCs, healthy controls; MNI, Montreal Neurological Institute; MB: monocular blindness.

### Analysis of receiver operating characteristic curves

**FIGURE 2:**
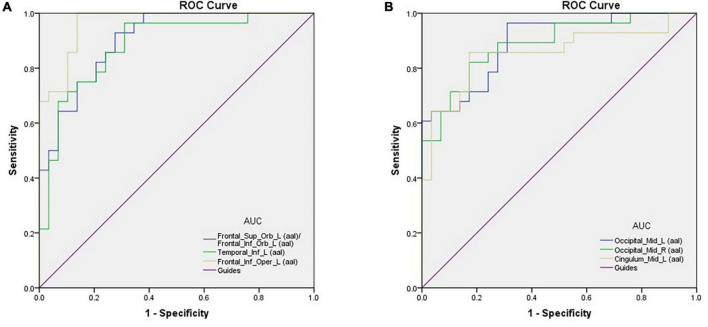
ROC curve analysis of the mean perAF values for altered brain regions. **(A)** The area under the ROC curve was 0.904, (*p* < 0.0001; 95% CI: 0.830–0.978) for Frontal_Sup_Orb_L (aal)/Frontal_Inf_Orb_L (aal); Temporal_Inf_L (aal) 0.883, (*p* < 0.0001; 95% CI: 0.794–0.972); Frontal_Inf_Oper_L (aal) 0.964, (*p* < 0.0001; 95% CI: 0.924–1.000). **(B)** The area under the ROC curve was 0.893 (*p* < 0.0001; 95% CI: 0.812–0.973) for Occipital_Mid_L (aal); Occipital_Mid_R (aal) 0.887, (*p* < 0.0001; 95% CI: 0.802–0.971); Cingulum_Mid_L (aal) 0.855, (*p* < 0.0001; 95% CI: 0.750–0.960). AUC, area under the curve; ROC, receiver operating characteristic.

Area under the curve provides an indication of diagnostic accuracy. AUC ranges from 0 to 1, higher values indicating higher accuracy. The AUC for brain regions defined here were between 0.86 and 0.96 and all were statistically significant (<0.0001) (Figure 2).

### Correlation analysis

**FIGURE 3:**
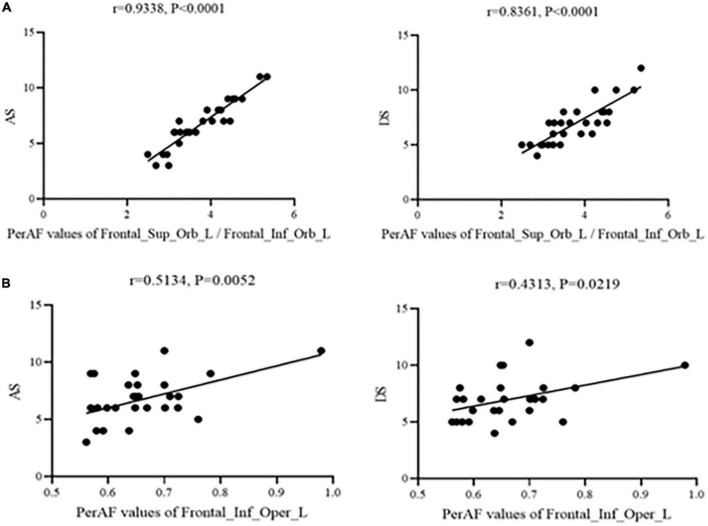
Correlation between PerAF and HADS scores. **(A)** Stands for monocular blindness group and **(B)** stands for healthy control group. In the MB group, the PerAF value of Frontal_Sup_Orb_L/Frontal_Inf_Orb_L showed a positive correlation with AS (*r* = 0.9338, *p* < 0.0001) and DS (*r* = 0.8361, *p* < 0.0001), and the value of Frontal_Inf_Oper_L also showed a positive correlation with AS (*r* = 0.5134, *p* < 0.05) and DS (*r* = 0.4313, *p* < 0.05). PerAF, percent amplitude of fluctuation; AS, anxiety scores; DS, depressed scores; MB, monocular blindness.

Figure 3 shows that correlation between PerAF values and HADS scores were significant at Frontal_Sup_Orb_L/Frontal_Inf_Orb_L for AS (*r* = 0.9338, *p* < 0.0001) and DS (*r* = 0.8361, *p* < 0.0001). Similarly, PerAF values at the Frontal_Inf_Oper_L were significantly positively correlated with AS (*r* = 0.5134, *p* < 0.05) and DS (*r* = 0.4313, *p* < 0.05) (Figure 3).

## Discussion

**FIGURE 4:**
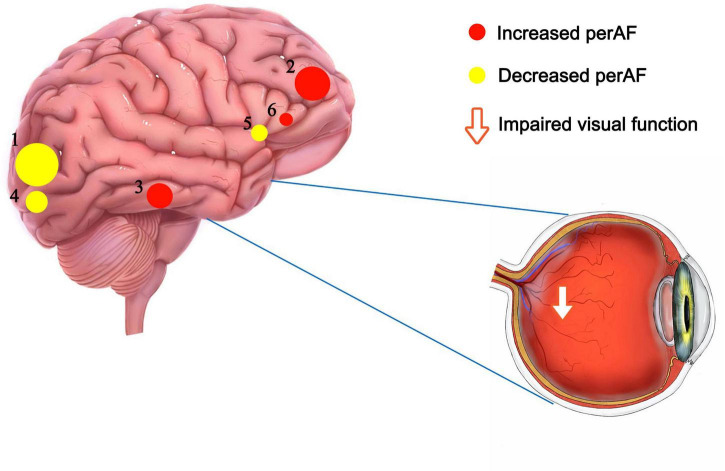
The mean perAF values of altered brain regions. Notes: Compared with the HCs, the perAF values of the following regions were increased to various extents: 2- Frontal_Sup_Orb_L/Frontal_Inf_Orb_L (t = -5.03), 3- Temporal_Inf_L (t = -4.92), 6- Frontal_Inf_Oper_L (t = -4.41). Compared with the HCs, the perAF values of the following regions were decreased to various extents: 1- Occipital_Mid_L (t = 5.21), 4- Occipital_Mid_R (t = 4.595), 6- Cingulum_Mid_L (t = 4.531). HCs, healthy controls; BA, Brodmann’s area.

In this study, the PerAF method was used to increase understanding of MB, and to our knowledge this is the first study in which MB has been investigated using this approach. The method is widely used and has also been applied to study other diseases (32–34) (Table 3). Our results showed that the signal values of Frontal_Sup_ Orb_L/Frontal_Inf_Orb_L/Temporal_Inf_L/Frontal_Inf_Oper _L regions are higher in MB patients than controls, while conversely signals are lower than controls at Occipital_Mid_L/Occipital_Mid_R/Cingulum_Mid_L (Figure 4 and Table 3).

**TABLE 3:** PerAF method applied in ophthalmologic and neurogenic disease.

Brain areas	Experimental results	Brain functions	Anticipated results
Frontal_Sup_Orb_L/Frontal_Inf_Orb_L	HC < MB	Emotion and depression, economic decisions, rewarding learning, decision making, alcohol abuse and dependence	Emotion problems, disability in dealing with daily tasks, social problems
Temporal_Inf_L	HC < MB	visual perception, multi-mode sensory integration	Cognitive impairment, mental disorder
Frontal_Inf_Oper_L	HC < MB	Reflect-Self contrast, the guidance of intonation processing, distinguishing concrete concepts from abstract concepts, creativity	Semantic comprehension disorder, conceptual comprehension disorder
Occipital_Mid_L	HC > MB	Visual information processing, attention, emotional processing, verbal episodic memory,	Depression, affective dysfunction, mental problems, memory problems
Occipital_Mid_R	HC > MB	visual spatial information processing, attention, working memory	Spatial vision problems, attention problems, memory disorder,
Cingulum_Mid_L	HC > MB	Social cognition, emotion processing, motor control, maturity	Emotion problems, cognition dysfunction, motor control disorder, maturational delay

The results of correlation analyses showed that in Frontal_Sup_Orb_L/Frontal_Inf_Orb_L/Frontal_Inf_Oper_L, AS and DS were positively correlated with PerAF values. Higher HADS scores indicate more severe levels of anxiety or depression, so this result indicates deeper anxiety and depression with higher PerAF values.

The orbitofrontal cortex (OFC) is an area of the brain in front of the eyes, consisting of a large cortical region on the ventral side of the frontal lobe (35). The OFC includes the orbital superior frontal gyrus and orbital inferior frontal gyrus, and it receives input from the visual, somatosensory, olfactory and taste regions, the limbic region, and the dorsal raphe region (36, 37). Rolls et al. reported that the OFC is related to emotion and depression (38), and that OFC plays an important role in day-to-day transactions (5). Izquierdo et al. conducted an animal study and found that OFC is associated with reward for learning and decision making (39). Other research has shown that the OFC is associated with alcohol abuse and dependence (40). In the present study, PerAF values were increased in the Frontal_Sup_Orb_L/Frontal_Inf_Orb_L regions in MB patients, indicating hyperactivity of this brain region. We infer that MB may be associated with difficulties related to emotion and social ability.

PerAF was also increased in the Temporal_Inf_L of MB patients. This region is situated on the lateral and inferior surfaces of the temporal lobe, ventral to the middle temporal gyrus (41). Previous research has shown that it participates in multiple cognitive processes, such as visual perception and multi-mode sensory integration (42–44). Onitsuka et al. reported the inferior temporal gyrus is fundamental to the pathophysiology of cognitive impairments in Alzheimer’s disease (41). In the present study, increased activity in this brain region suggests that a range of cognitive anomalies may occur in MB patients.

A study reported that the left inferior frontal cortex has an influence on reflect-Self contrast (45), and has a role in the guidance of intonation processing (46), Other research findings have shown that this region may be viewed as a neural intersection for different types of information, and is important for distinguishing between concrete and abstract concepts (47). Study (48) has shown that suppressing this region may allow activation of neural networks that lead to greater creativity. The left operculum of left inferior frontal cortex is associated with sensorimotor function, such as the experience of pain (49). Since the PerAF value in this region is higher in MB patients than in HCs, we hypothesize that the risk of disease associated with dysfunction in this region may be increased in this group.

The occipital lobe, which takes up most of the visual cortex, helps with the processing of visual information and plays a role in exclamatory facial expressions, and in this study, it turned out that left middle occipital may be associated with depression in women (50), moreover, the region is also involved in attention (51), verbal episodic memory (52), and affective dysfunction (53), and Stern et al. (53) found that in adults with obsessive-compulsive disorder, spontaneous activity in this region is increased. In contrast to the brain regions discussed above, the decreased PerAF signal values in the left middle occipital in MB patients compared with HCs indicates that this brain region is functionally impaired in MB patients.

Similar to the left middle occipital, the right middle occipital lobe is associated with visual spatial information (54) and attention (55). Zeng et al. (56) found that function of the right middle occipital was positively correlated with object working memory. On the right side of the middle occipital gyrus, we observed decreased brain activity in MB patients, indicating that the function of this area was reduced.

Finally, we found a decrease in brain activity in the left middle cingulum in MB patients. The cingulate gyrus belongs to the medial cortex and medial temporal lobe (57), and plays an important role in social cognition (58), emotional processing (59) and motor control (60). A study on attention disorder/hyperactivity disorder found dysfunction of the left middle cingulum in MB patients, which was attributed to delayed maturation (61). We infer that the abnormal spontaneous activity of this brain region in MB patients may reflect abnormality of functions related to this region (Table 4).

**TABLE 4:** Brain areas alternation and its potential functions. HC, healthy controls; MB, monocular blindness.

Brain areas				
Author, year	Disease	UDs > HCs UDs < HCs		(Refs.)
Yang et al. (32)	Retinal detachment	Right fusiform gyrus, left inferior temporal gyrus,		(32)
Wang et al. (33)	Epilepsy	Vermis, left cellebellar lobule, left percentral gyrus	Pecentral gyrus	(33A
Zeng et al. (34)	Sleep deprivation	Bilateral visual cortex, bilateral sensorimotor cortex	Bilateral dorsolateral prefrontal cortex, bilateral cerebellum posterior lobe	(34)

### Limitations

This study included a small sample, which may not be representative of the wider population of patients with MB. In addition, the included MB patients had a range of durations since diagnosis of MB, which may have increased variance in the experimental results. Next, we will increase the sample size, conduct multi sequence analysis, follow-up patients, explore ways to treat brain function and try to make up for deficiencies.

## Conclusion

In this study, we used the PerAF method to analyze regional brain activity in MB patients. Compared with healthy controls, hyperactivity in some brain regions and hypoactivity in other regions may be related to anomalous function and behavior associated with these brain regions. To the best of our knowledge, this is the first study on MB using the PerAF method. Future studies of this kind may further enhance understanding of neural changes in MB and may lead to the use of this method as an early diagnostic index.

## Data availability statement

The original contributions presented in this study are included in the article/supplementary material, further inquiries can be directed to the corresponding author.

## Ethics statement

The studies involving human participants were reviewed and approved by Medical Ethics Committee of The First Affiliated Hospital of Nanchang University (Nanchang, China). The patients/participants provided their written informed consent to participate in this study.

## Author contributions

QH, JC, and MK analyzed the data and draft the manuscript. PY, XL, and JZ assisted with data interpretation and figure composing. TS, YW, and HW collected the data. YS conceived, designed, and directed the study, final revised, and approved the manuscript. All authors contributed to the article and approved the submitted version.

## Footnotes

1
http://rfmri.org/DPARSF

## References

[1] Vashist P Senjam SS Gupta V Gupta N Kumar A. Definition of blindness under National Programme for Control of Blindness: do we need to revise it? *Indian J Ophthalmol.* (2017) 65:92–6. 10.4103/ijo.IJO_869_16 28345562 PMC5381306

[2] Fisher CR Ferrington DA. Perspective on AMD pathobiology: a bioenergetic crisis in the RPE. *Invest Ophthalmol Vis Sci.* (2018) 59:AMD41–7. 10.1167/iovs.18-24289 30025108 PMC5989860

[3] Lee CM Afshari NA. The global state of cataract blindness. *Curr Opin Ophthalmol.* (2017) 28:98–103. 10.1097/ICU.0000000000000340 27820750

[4] Silva EJD Pereira DP Ambrózio JOAM Barboza LM Fonseca VL Caldeira AP. Prevalence of trachoma and associated factors in students from the Jequitinhonha Valley, Minas Gerais, Brazil. *Rev Soc Bras Med Trop.* (2020) 53:e20200056. 10.1590/0037-8682-0056-2020 33111907 PMC7580275

[5] Hu YX Xu XX Shao Y Yuan GL Mei F Zhou Q The prognostic value of lymphocyte-to-monocyte ratio in retinopathy of prematurity. *Int J Ophthalmol.* (2017) 10:1716–21. 10.18240/ijo.2017.11.13 29181316 PMC5686371

[6] Sahel JA Bennett J Roska B. Depicting brighter possibilities for treating blindness. *Sci Transl Med.* (2019) 11:eaax2324. 10.1126/scitranslmed.aax2324 31142676

[7] Bourne RRA Flaxman SR Braithwaite T Cicinelli MV Das A Jonas JB Magnitude, temporal trends, and projections of the global prevalence of blindness and distance and near vision impairment: a systematic review and meta-analysis. *Lancet Glob Health.* (2017) 5:e888–97. 10.1016/S2214-109X(17)30293-0 28779882

[8] Richard AI. Monocular blindness in Bayelsa state of Nigeria. *Pan Afr Med J.* (2010) 4:6. 10.4314/pamj.v4i1.53607 21119991 PMC2984307

[9] Jones RK Lee DN. Why two eyes are better than one: the two views of binocular vision. *J Exp Psychol Hum Percept Perform.* (1981) 7:30–40. 10.1037//0096-1523.7.1.30 6452501

[10] Bansal RK Khandekar R Nagendra P Kurup P. Magnitude and causes of unilateral absolute blindness in a region of Oman: a hospital-based study. *Eur J Ophthalmol.* (2007) 17:418–23. 10.1177/112067210701700325 17534827

[11] Eballe AO Epée E Koki G Bella L Mvogo CE. Unilateral childhood blindness: a hospital-based study in Yaoundé, Cameroon. *Clin Ophthalmol.* (2009) 3:461–4. 10.2147/opth.s5289 19714264 PMC2732056

[12] Nwosu SN. Blindness and visual impairment in Anambra State, Nigeria. *Trop Geogr Med.* (1994) 46:346–9. 7892700

[13] Huang X Li D Li HJ Zhong YL Freeberg S Bao J Abnormal regional spontaneous neural activity in visual pathway in retinal detachment patients: a resting-state functional MRI study. *Neuropsychiatr Dis Treat.* (2017) 13:2849–54. 10.2147/NDT.S147645 29200859 PMC5703148

[14] Harrell J Larson ND Menza E Mboti AA. Clinic-based survey of blindness in Kenya. *Community Eye Health.* (2001) 14:68–9. 17491939 PMC1705942

[15] Buch H Vinding T La Cour M Nielsen NV. The prevalence and causes of bilateral and unilateral blindness in an elderly urban Danish population. The copenhagen city eye study. *Acta Ophthalmol Scand.* (2001) 79:441–9. 10.1034/j.1600-0420.2001.790503.x 11594976

[16] Brown HD Woodall RL Kitching RE Baseler HA Morland AB. Using magnetic resonance imaging to assess visual deficits: a review. *Ophthalmic Physiol Opt.* (2016) 36:240–65. 10.1111/opo.12293 27112223 PMC4855621

[17] Evans SL Dowell NG Prowse F Tabet N King SL Rusted JM. Mid age APOE ε4 carriers show memory-related functional differences and disrupted structure-function relationships in hippocampal regions. *Sci Rep.* (2020) 10:3110. 10.1038/s41598-020-59272-0 32080211 PMC7033211

[18] Yang J Xiong J Yuan T Wang X Jiang Y Zhou X Effectiveness and safety of acupuncture and moxibustion for primary dysmenorrhea: an overview of systematic reviews and meta-analyses. *Evid Based Complement Alternat Med.* (2020) 2020:8306165. 10.1155/2020/8306165 32419829 PMC7206866

[19] Ogawa S Menon RS Kim SG Ugurbil K. On the characteristics of functional magnetic resonance imaging of the brain. *Annu Rev Biophys Biomol Struct.* (1998) 27:447–74. 10.1146/annurev.biophys.27.1.447 9646874

[20] Huang X Li HJ Ye L Zhang Y Wei R Zhong YL Altered regional homogeneity in patients with unilateral acute open-globe injury: a resting-state functional MRI study. *Neuropsychiatr Dis Treat.* (2016) 12:1901–6. 10.2147/NDT.S110541 27536111 PMC4975161

[21] Jia XZ Sun JW Ji GJ Liao W Lv YT Wang J Percent amplitude of fluctuation: a simple measure for resting-state fMRI signal at single voxel level. *PLoS One.* (2020) 15:e0227021. 10.1371/journal.pone.0227021 31914167 PMC6948733

[22] Li T Liu Z Li J Liu Z Tang Z Xie X Altered amplitude of low-frequency fluctuation in primary open-angle glaucoma: a resting-state FMRI study. *Invest Ophthalmol Vis Sci.* (2014) 56:322–9. 10.1167/iovs.14-14974 25525176

[23] Pan ZM Li HJ Bao J Jiang N Yuan Q Freeberg S Altered intrinsic brain activities in patients with acute eye pain using amplitude of low-frequency fluctuation: a resting-state fMRI study. *Neuropsychiatr Dis Treat.* (2018) 14:251–7. 10.2147/NDT.S150051 29386898 PMC5767092

[24] Shao Y Cai FQ Zhong YL Huang X Zhang Y Hu PH Altered intrinsic regional spontaneous brain activity in patients with optic neuritis: a resting-state functional magnetic resonance imaging study. *Neuropsychiatr Dis Treat.* (2015) 11:3065–73. 10.2147/NDT.S92968 26715848 PMC4686319

[25] Wang ZL Zou L Lu ZW Xie XQ Jia ZZ Pan CJ. Abnormal spontaneous brain activity in type 2 diabetic retinopathy revealed by amplitude of low-frequency fluctuations: a resting-state fMRI study. *Clin Radiol.* (2017) 72:340.e1–7. 10.1016/j.crad.2016.11.012 28041652

[26] Wu YY Yuan Q Li B Lin Q Zhu PW Min YL Altered spontaneous brain activity patterns in patients with retinal vein occlusion indicated by the amplitude of low-frequency fluctuation: a functional magnetic resonance imaging study. *Exp Ther Med.* (2019) 18:2063–71. 10.3892/etm.2019.7770 31410162 PMC6676080

[27] Zuo XN Di Martino A Kelly C Shehzad ZE Gee DG Klein DF The oscillating brain: complex and reliable. *Neuroimage.* (2010) 49:1432–45. 10.1016/j.neuroimage.2009.09.037 19782143 PMC2856476

[28] Zhao N Yuan LX Jia XZ Zhou XF Deng XP He HJ Intra- and inter-scanner reliability of voxel-wise whole-brain analytic metrics for resting state fMRI. *Front Neuroinform.* (2018) 12:54. 10.3389/fninf.2018.00054 30186131 PMC6110941

[29] Yang L Yan Y Wang Y Hu X Lu J Chan P Gradual disturbances of the Amplitude of low-frequency fluctuations (ALFF) and fractional ALFF in Alzheimer spectrum. *Front Neurosci.* (2018) 12:975. 10.3389/fnins.2018.00975 30618593 PMC6306691

[30] Chao-Gan Y Yu-Feng Z. DPARSF: a MATLAB toolbox for “pipeline” data analysis of resting-state fMRI. *Front SystNeurosci.* (2010) 4:13. 10.3389/fnsys.2010.00013 20577591 PMC2889691

[31] Fox MD Snyder AZ Vincent JL Corbetta M Van Essen DC Raichle ME. The human brain is intrinsically organized into dynamic, anticorrelated functional networks. *Proc Natl Acad Sci U.S.A.* (2005) 102:9673–8. 10.1073/pnas.0504136102 15976020 PMC1157105

[32] Yang YC Li QY Chen MJ Zhang LJ Zhang MY Pan YC Investigation of changes in retinal detachment-related brain region activities and functions using the percent amplitude of fluctuation method: a resting-state functional magnetic resonance imaging study. *Neuropsychiatr Dis Treat.* (2021) 17:251–60. 10.2147/NDT.S292132 33536757 PMC7850567

[33] Wang B Wang J Cen Z Wei W Xie F Chen Y Altered cerebello-motor network in familial cortical myoclonic tremor with epilepsy type 1. *Mov Disord.* (2020) 35:1012–20. 10.1002/mds.28014 32129927

[34] Zeng B Zhou J Li Z Zhang H Li Z Yu P. Altered percent amplitude of fluctuation in healthy subjects after 36 h sleep deprivation. *Front Neurol.* (2021) 11:565025. 10.3389/fneur.2020.565025 33519662 PMC7843545

[35] Rudebeck PH Rich EL. Orbitofrontal cortex. *Curr Biol.* (2018) 28:R1083–8. 10.1016/j.cub.2018.07.018 30253144 PMC9253859

[36] Ongür D Price JL. The organization of networks within the orbital and medial prefrontal cortex of rats, monkeys and humans. *Cereb Cortex.* (2000) 10:206–19. 10.1093/cercor/10.3.206 10731217

[37] Lv J Chen Q Shao Y Chen Y Shi J. Cross-talk between angiotensin-II and toll-like receptor 4 triggers a synergetic inflammatory response in rat mesangial cells under high glucose conditions. *Biochem Biophys Res Commun.* (2015) 459:264–9. 10.1016/j.bbrc.2015.02.096 25732086

[38] Rolls ET. The orbitofrontal cortex and emotion in health and disease, including depression. *Neuropsychologia.* (2019) 128:14–43. 10.1016/j.neuropsychologia.2017.09.021 28951164

[39] Izquierdo A. Functional heterogeneity within rat orbitofrontal cortex in reward learning and decision making. *J Neurosci.* (2017) 37:10529–40. 10.1523/JNEUROSCI.1678-17.2017 29093055 PMC6596524

[40] Moorman DE. The role of the orbitofrontal cortex in alcohol use, abuse, and dependence. *Prog Neuropsychopharmacol Biol Psychiatry.* (2018) 87(Pt. A):85–107. 10.1016/j.pnpbp.2018.01.010 29355587 PMC6072631

[41] Onitsuka T Shenton ME Salisbury DF Dickey CC Kasai K Toner SK Middle and inferior temporal gyrus gray matter volume abnormalities in chronic schizophrenia: an MRI study. *Am J Psychiatry.* (2004) 161:1603–11. 10.1176/appi.ajp.161.9.1603 15337650 PMC2793337

[42] Ishai A Ungerleider LG Martin A Schouten JL Haxby JV. Distributed representation of objects in the human ventral visual pathway. *Proc Natl Acad Sci U.S.A.* (1999) 96:9379–84. 10.1073/pnas.96.16.9379 10430951 PMC17791

[43] Herath P Kinomura S Roland PE. Visual recognition: evidence for two distinctive mechanisms from a PET study. *Hum Brain Mapp.* (2001) 12:110–9. 10.1002/1097-0193(200102)12:23.0.co;2-0 11169875 PMC6871813

[44] Mesulam MM. From sensation to cognition. *Brain.* (1998) 121 (Pt. 6):1013–52. 10.1093/brain/121.6.1013 9648540

[45] McAdams CJ Harper JA Van Enkevort E. Mentalization and the left inferior frontal gyrus and insula. *Eur Eat Disord Rev.* (2018) 26:265–71. 10.1002/erv.2580 29464819 PMC5965288

[46] van der Burght CL Goucha T Friederici AD Kreitewolf J Hartwigsen G. Intonation guides sentence processing in the left inferior frontal gyrus. *Cortex.* (2019) 117:122–34. 10.1016/j.cortex.2019.02.011 30974320

[47] Della Rosa PA Catricalà E Canini M Vigliocco G Cappa SF. The left inferior frontal gyrus: a neural crossroads between abstract and concrete knowledge. *Neuroimage.* (2018) 175:449–59. 10.1016/j.neuroimage.2018.04.021 29655937

[48] Kleinmintz OM Abecasis D Tauber A Geva A Chistyakov AV Kreinin I Participation of the left inferior frontal gyrus in human originality. *Brain Struct Funct.* (2018) 223:329–41. 10.1007/s00429-017-1500-5 28828749

[49] Garcia-Larrea L. The posterior insular-opercular region and the search of a primary cortex for pain. *Neurophysiol Clin.* (2012) 42:299–313. 10.1016/j.neucli.2012.06.001 23040701

[50] Ouyang J Yang L Huang X Zhong YL Hu PH Zhang Y The atrophy of white and gray matter volume in patients with comitant strabismus: evidence from a voxel-based morphometry study. *Mol Med Rep.* (2017) 16:3276–82. 10.3892/mmr.2017.7006 28713925 PMC5547961

[51] Roxo MR Franceschini PR Zubaran C Kleber FD Sander JW. The limbic system conception and its historical evolution. *Sci World J.* (2011) 11:2428–41. 10.1100/2011/157150 22194673 PMC3236374

[52] Li G Ma X Bian H Sun X Zhai N Yao M A pilot fMRI study of the effect of stressful factors on the onset of depression in female patients. *Brain Imaging Behav.* (2016) 10:195–202. 10.1007/s11682-015-9382-8 25864196 PMC4781893

[53] Shao Y Yu Y Pei CG Tan YH Zhou Q Yi JL Therapeutic efficacy of intracameral amphotericin B injection for 60 patients with keratomycosis. *Int J Ophthalmol.* (2010) 3:257–60. 10.3980/j.issn.2222-3959.2010.03.18 22553567 PMC3340624

[54] Bristow D Frith C Rees G. Two distinct neural effects of blinking on human visual processing. *Neuroimage.* (2005) 27:136–45. 10.1016/j.neuroimage.2005.03.037 15893941

[55] Thakral PP Slotnick SD. The role of parietal cortex during sustained visual spatial attention. *Brain Res.* (2009) 1302:157–66. 10.1016/j.brainres.2009.09.031 19765554

[56] Ren Z Zhang Y He H Feng Q Bi T Qiu J. The different brain mechanisms of object and spatial working memory: voxel-based morphometry and resting-state functional connectivity. *Front Hum Neurosci.* (2019) 13:248. 10.3389/fnhum.2019.00248 31379543 PMC6659551

[57] Hau J Aljawad S Baggett N Fishman I Carper RA Müller RA. The cingulum and cingulate U-fibers in children and adolescents with autism spectrum disorders. *Hum Brain Mapp.* (2019) 40:3153–64. 10.1002/hbm.24586 30941791 PMC6865481

[58] Amodio DM Frith CD. Meeting of minds: the medial frontal cortex and social cognition. *Nat Rev Neurosci.* (2006) 7:268–77. 10.1038/nrn1884 16552413

[59] Bush G Luu P Posner MI. Cognitive and emotional influences in anterior cingulate cortex. *Trends Cogn Sci.* (2000) 4:215–22. 10.1016/s1364-6613(00)01483-2 10827444

[60] Shao Y Bao J Huang X Zhou FQ Ye L Min YL Comparative study of interhemispheric functional connectivity in left eye monocular blindness versus right eye monocular blindness: a resting-state functional MRI study. *Oncotarget.* (2018) 9:14285–95. 10.18632/oncotarget.24487 29581843 PMC5865669

[61] Zhao Y Cui D Lu W Li H Zhang H Qiu J. Aberrant gray matter volumes and functional connectivity in adolescent patients with ADHD. *J Magn Reson Imaging.* (2020) 51:719–26. 10.1002/jmri.26854 31265198

